# COVID-19 Severity among Healthcare Workers: Overweight Male Physicians at Risk

**DOI:** 10.3390/idr14030036

**Published:** 2022-04-25

**Authors:** Bahar Madran, Zeliha Akbulut, Gözde Akbaba, Emre Taş, Tuğba Güçlüoğlu, Özgür Şencanlı, İsmail Bozkurt, Şiran Keske, Önder Ergönül

**Affiliations:** 1 Infection Control Unit, VKV American Hospital, Istanbul 34365, Turkey; gozdea@amerikanhastanesi.org; 2 Infection Control Unit, Koç University Hospital, Istanbul 34010, Turkey; zakbulut@kuh.ku.edu.tr (Z.A.); etas@kuh.ku.edu.tr (E.T.); tzengin@kuh.ku.edu.tr (T.G.); 3 Occupational Safety Unit, VKV American Hospital, Istanbul 34365, Turkey; ozgurs@amerikanhastanesi.org; 4 Hospital Management Department, VKV American Hospital, Istanbul 34365, Turkey; ismailb@amerikanhastanesi.org; 5 Infectious Diseases and Clinical Microbiology, School of Medicine, Koç University, Istanbul 34010, Turkey; skeske@ku.edu.tr (Ş.K.); oergonul@ku.edu.tr (Ö.E.); 6 İşbank Center for Infectious Diseases, Koç University, Istanbul 34010, Turkey

**Keywords:** COVID-19, healthcare workers, pandemic, severity predictors

## Abstract

We performed a prospective longitudinal cohort study in two healthcare settings. In total, 909 HCWs out of 3982 (23.35%) were diagnosed with COVID-19 before the vaccination era. Eighty-five per cent of COVID-19 positive HCWs (n = 774) were asymptomatic or mild, and 15% were moderate or severe. The mean age of the infected HCWs in the moderate or severe group was higher than the mild or asymptomatic group (35.4 vs. 31.3 years, *p* < 0.001). Thirty-two per cent of HCWs were male and the rate of male gender was more frequent in the moderate/severe group (*p* = 0.009). The rate of those who have cardiovascular diseases (*p* = 0.003) and diabetes mellitus (*p* = 0.044) were significantly higher among the HCWs with moderate or severe COVID-19. In multivariate analysis, male gender (OR:1.65, CI:1.11–2.46, *p* = 0.013), BMI > 30 (OR: 1.9, CI: 1.09–3.51, *p* = 0.024), and being physician (OR: 2.56, CI:1.45–4.52, *p* = 0.001) were found to be associated with moderate or severe COVID-19.

## 1. Introduction

Healthcare workers (HCWs) were reported to have a higher risk of SARS-CoV-2 infection because of repetitive, close, and prolonged contact with COVID-19 patients [1,2]. The WHO has reported that 6645 HCWs died due to the SARS-CoV-2 infection between January 2020 and May 2021 [3]. Occupational exposure to the SARS-CoV-2 virus increases the risk of infection among HCWs, particularly lack of personal protective equipment, asymptomatic carriers, and presenteeism in the hospital have been associated with the spread of SARS-CoV-2 [4]. In addition to occupational exposure, we described the significance of community-acquired infection rates in our previous study [5]. Moreover, it was reported that COVID-19 was seven times more severe among the HCWs compared to the community [4], and we would like to describe this point based on our data. Several underlying conditions have a significant independent effect on the severity of COVID-19. Advanced age, being male, having comorbidities, obesity, smoking, and pregnancy were reported to be predictive risk factors of COVID-19 severity among the general population [6].

The HCWs make a convenient cohort for observation of the disease course besides the transmission dynamics [7]. Detailed analysis of the clinical course of HCWs will also provide information about the management of the disease. In this study, we aimed to describe the severity level of the SARS-CoV-2 infection among HCWs, and compare the risk factors defined for the community.

## 2. Materials and Methods

We performed a prospective longitudinal cohort study in two hospitals in Istanbul. All HCWs who have any clinical symptoms of COVID-19 (fever, cough, respiratory symptoms, headaches, myalgia, anosmia, ageusia) have been monitored by the occupational health and safety departments (OHSD) of each hospital since the first COVID-19 case was detected in the hospital. Before the vaccination period, the HCWs were included from 11 March 2020, until 11 March 2021. The institutional review board of Koç University approved the study (2020.216.IRB1.066).

We included all the HCWs who were diagnosed with COVID-19 by hospital OHSD. The information about the epidemiology, clinical symptoms, medical history, laboratory results, stand length, and vaccination of hospital workers was collected from electronic medical records and OHSD. All the HCWs were followed-up daily by the nurse of the OHSD unit. All COVID-19 positive HCWs were diagnosed and classified according to the CDC Guideline [8].

Statistical analysis was performed by using STATA 14v (Texas, TX, USA). For categorical variables—chi-square, for continuous variables—*t*-test was used. In multivariate analysis for predicting moderate or severe cases, logistic regression with backward selection was performed. Dependent variables included in the model were gender, age > 40, BMI > 30, physician, and cardiovascular diseases. Statistical significance was set as *p* < 0.05.

## 3. Results

In two hospitals, 909 HCWs out of 3982 (23.35%) were diagnosed with COVID-19 (Table 1) before the vaccination period between 11 March 2020, and 10 March 2021. Eighty-five per cent of COVID-19 positive HCWs (n = 774) were asymptomatic or mild, and 15% were moderate or severe. None of the HCWs needed ventilation support, and none died because of COVID-19 disease.

The mean age of the infected HCWs in the moderate or severe group was higher than in the mild or asymptomatic group (35.4 vs. 31.3 years, *p* < 0.001, Table 1), accordingly in the moderate or severe group, the proportion of HCWs > 40 years of age was more than the HCWs in the asymptomatic or mild group (*p* = 0.006). Thirty-two per cent of HCWs were male and the rate of male gender was more frequent in the moderate/severe group (*p* = 0.009, Table 1). The rate of physicians among the asymptomatic and mild groups was 7%, and 16% among the moderate and severe groups (*p* < 0.001, Table 1). Forty-five HCWs had comorbidities such as cardiovascular diseases or diabetes mellitus (Table 1). The rate of those who have cardiovascular diseases (*p* = 0.003) and diabetes mellitus (*p* = 0.044) were significantly higher among the HCWs with moderate or severe COVID-19.

Two hundred sixty-four positive HCWs were reported to have household contact with symptomatic or asymptomatic family members, 229 positive HCWs have a history of communication with other positive HCWs, 103 HCWs were exposed to patients with inappropriate personal protective equipment (PPE), and 303 positive HCWs has not indicated any contact description (Table 1). The inappropriate use of PPE was higher among the moderate or severe group than in the asymptomatic or mild group (*p* = 0.010).

In multivariate analysis, male gender (OR: 1.65, CI:1.11–2.46, *p* = 0.013), BMI > 30 (OR: 1.9, CI: 1.09–3.51, *p* = 0.024), and being physician (OR: 2.56, CI: 1.45–4.52, *p* = 0.001) were found to be associated with moderate or severe COVID-19 (Table 2).

## 4. Discussion

In this study, we focused on the predictors of disease severity in COVID-19 among HCWs. In a multivariate analysis, being male, being a physician, and having BMI > 30 were detected to be independent risk factors for severe COVID-19 infection among HCWs. However, in a univariate analysis, age of >40 years and having cardiovascular disease were detected to be significant risk factors. In previous studies, diabetes mellitus, hypertension, male gender, being older, obesity, and cardiovascular diseases were reported as risk factors for the severity of COVID-19 [9,10,11].

Being male and advanced age were reported as independent severity factors in the community [12,13,14], and also among healthcare workers. In previous studies, the infection rate was reported to be higher among female (72%) HCWs than male HCWs (28%), while the case fatality rate among females (29%) was lower than male HCWs (71%) [15]. In the same study, it was reported that the infection rate among nurses (38%) was higher than physicians, however, physicians’ mortality rate was higher than nurses’ (51% and 25%) [15]. In parallel to these findings, in our study group, the infection rate among physicians was found to be 12.8% (77 out of 594) and 20.7% among the nurses (258 out of 1243, *p* = 0.001). There was no fatality in the study group, however, disease severity was higher among the physicians.

At the beginning of the pandemic, it was reported that the SARS-CoV-2 infection could be more severe among the HCWs than in the community, and this was one of the aims of our study. In a recent systematic review, severe clinical complications developed in 5% of the COVID-19 positive HCWs and 0.5% (95% CI; 0.02–1.3%) of them died [2]. These findings were supported by a cohort study, which showed that the mortality rate of the HCWs was not higher than the general population [16]. The HCWs as an actively working group of people might have fewer comorbidities and lack advanced age compared to the community, therefore the rate of severity and fatality among the HCWs would be expected to be less than the community.

In terms of transmission route, 264 (29%) positive HCWs reported household contact with symptomatic or asymptomatic family members. In one of our previous studies, we reported that medical secretaries and janitorial staff had a higher rate of infections, and they acquired the infection out of the hospital [17]. The rate of household contact was not significantly different between asymptomatic/mild and moderate/severe cases (*p* = 0.638). Two hundred twenty-nine positive (28%) HCWs have a contact history with other infected HCWs. One hundred-three (11%) HCWs have reported exposure to a positive patient with inappropriate PPE. This rate may change according to the overload of the COVID-19 patients in the hospital, the knowledge gap of the HCWs, and the resources of the hospital [18,19]. Some patients were detected after hospital admission, therefore HCWs might be exposed with loose precautions. Another reason for using inappropriate PPE was using a surgical face mask instead of a respirator mask during an aerosol-generating procedure if the patient’s COVID-19 diagnosis is unclear [5]. The improper use of PPE was significantly higher among moderate or severe HCWs than in asymptomatic or mild ones (*p* = 0.010). The inappropriate use of PPE not only causes the transmission of infection but also increases exposure to higher viral load that might affect the severity of the infection.

For our study aim, our study has a limited sample size to represent all the HCWs, however the strength of our study is to be prospective and have complete data for precise analysis.

## 5. Conclusions

Being male, being a physician, and having BMI > 30 were independent risk factors for severe COVID-19 among HCWs. The rate of severity among HCWs is not higher than in the community. The OHSDs could increase the awareness of the HCWs with defined risk groups for severe infection by alerting them at the beginning of the diagnosis.

## Figures and Tables

**Table 1:** Univariate analysis for risk factors of COVID-19 severity.

	Total n = 909 (%)	Asymptomatic or Mild n = 774 (%)	Moderate or Severe n = 135 (%)	*p*
Mean age (SD; min-max)	31.9 (8.8; 18–72)	31.3 (8.5; 18–62)	35.4 (9.8; 21–72)	<0.001
Age > 40	236 (26)	188 (24)	48 (36)	0.006
Gender				
Female	620 (68)	541 (70)	79 (58)	0.009
Male	289 (32)	233 (30)	56 (41)	
Occupation				
Physician	77 (8)	55 (7)	22 (16)	<0.001
Nurse	258 (28)	221 (29)	37 (27)	0.785
Porter	80 (9)	67 (9)	13 (10)	0.713
Secretary	72 (8)	63 (8)	9 (7)	0.559
Pharmacist	9 (1)	8 (1)	1 (1)	0.751
Technician	98 (11)	81 (10)	17 (13)	0.462
Support personnel	46	41 (89)	5 (11)	0.436
Others	269 (30)	243 (31)	26 (19)	
Comorbidity				
Cardiovascular diseases	34 (3.7)	23 (3)	11 (8)	0.003
Diabetes	11 (1.2)	7 (1)	4 (3)	0.044
Route of transmission				
Household	264 (29)	227 (29)	37 (27)	0.638
Other HCWs	229 (28)	193 (28)	36 (29)	0.699
Exposure to the patients with inappropriate PPE	103 (11)	79 (10)	24 (18)	0.010

**Table 2:** Predictors of severity among healthcare workers with COVID-19 (backward selection).

	Univariate			Multivariate		
	OR	CI	*p*	OR	CI	*p*
Male gender	1.64	1.13–2.39	0.009	1.65	1.11–2.46	0.013
Age > 40 years	1.71	1.16–2.52	0.006	-	-	-
BMI > 30 kg/m^2^	2	1.13–3.54	0.017	1.9	1.09–3.51	0.024
Cardiovascular diseases	2.89	1.37–6.09	0.005	-	-	-
Physician	2.54	1.49–4.33	0.001	2.56	1.45–4.52	0.001

## Data Availability

Not applicable.
